# Dominant optic atrophy in Denmark – report of 15 novel mutations in *OPA1*, using a strategy with a detection rate of 90%

**DOI:** 10.1186/1471-2350-13-65

**Published:** 2012-08-02

**Authors:** Gitte J Almind, Jakob Ek, Thomas Rosenberg, Hans Eiberg, Michael Larsen, LuCamp LuCamp, Karen Brøndum-Nielsen, Karen Grønskov

**Affiliations:** 1 Center for Applied Human Molecular Genetics, Kennedy Center, Glostrup, Denmark; 2 Department of Clinical Genetics, Rigshospitalet, Copenhagen, Denmark; 3 National Eye Clinic, Kennedy Center, Glostrup, Denmark; 4 Department of Cellular and Molecular Medicine, Faculty of Health Sciences, University of Copenhagen, Copenhagen, Denmark; 5 Department of Ophthalmology, Glostrup Hospital, Glostrup, Denmark; 6 LuCamp, TheLundbeck Foundation Centre for Applied Medical Genomics in Personalized Disease Prediction, Prevention and Care, Copenhagen, Denmark

**Keywords:** OPA1, Optic atrophy, Optic atrophies, Hereditary, Autosomal dominant, ADOA, Genetics, Optic neuropathies, Optic nerve, Genotype, Phenotype

## Abstract

**Background:**

Investigation of the *OPA1* mutation spectrum in autosomal dominant optic atrophy (ADOA) in Denmark.

**Methods:**

Index patients from 93 unrelated ADOA families were assessed for a common Danish founder mutation (c.2826_2836delinsGGATGCTCCA) in*OPA1*. If negative, direct DNA sequencing of the coding sequence and multiplex ligation-dependent probe amplification (MLPA) were performed. Results from MLPA analysis have been previously reported. Haplotype analysis was carried out analysing single nucleotide polymorphisms (SNP). Retrospective clinical data were retrieved from medical files.

**Results:**

Probably causative mutations were identified in 84 out of 93 families (90%) including 15 novel mutations. Three mutations c.983A > G, c.2708_2711delTTAG and c.2826_2836delinsGGATGCTCCA, were responsible for ADOA in10, 11 and 28 families, respectively, corresponding to 11%, 12% and 30%. A common haplotype in nine of ten c.983A > G families suggests that they descend from a single founder. The c.2708_2711delTTAG mutation was present on at least two haplotypes and has been repeatedly reported in various ethnic groups,thus represents a mutational hotspot. Clinical examinations of index patients with the two latter mutations demonstrated large inter- and intra-familial variations apparently.

**Conclusions:**

Genetic testing for *OPA1*mutations assist in the diagnosis. We have identified mutations in *OPA1* in 90% of families including 15 novel mutations. Both DNA sequencing and MLPA analysis are necessary to achieve a high detection rate. More than half of the affected families in Denmark are represented by three common mutations, at least two of which are due to a founder effect, which may account for the high prevalence of ADOA in Denmark.

## Background

Autosomal dominant optic atrophy (ADOA) is characterized by bilateral visual loss, preferential temporal disk pallor, dyschromatopsia, relative or absolute centrocoecalscotoma, and subnormal thickness of the retinal ganglion cell and nerve-fiber layers. The disease has incomplete penetrance and variable expressivity, ranging from subclinical visual dysfunction to legal blindness. The highly variable phenotype, both within and between families, suggests that modifier genes and/or environmental factors influence the expression of the disease. The prevalence of 1:12,000 in Denmark is remarkably high compared to 1:35,000 in other populations. [1,2] Monosymptomatic optic atrophy shows genetic heterogeneity with at least eight loci; *OPA1* (OMIM 605290) is the major ADOA gene. OPA2 (OMIM 311050) has been mapped to Xp11.4-p11.21, mutations in the *OPA3* gene (OMIM 606580) have been reported in ADOA associated with cataract (ADOAC) (OMIM 165300), [3] OPA4 (OMIM 605293) has been mapped to chromosome 18q12.2-q12.3, OPA5 (OMIM 610708) to chromosome 22q12.1-q13.1 and OPA6 (OMIM 258500) to chromosome 8q. OPA7 (OMIM 612989) is caused by mutations in *TMEM126A* (OMIM 612988). [4] Recently the OPA8 locus has been mapped to chromosome 16q21-q22 [5].

*OPA1*is located on chromosome 3q28-q29, [6,7] and more than 200 unique pathogenic mutations associated with Kjer-type optic atrophy (OMIM 165500) have been identified (http://lbbma.univ-angers.fr/eOPA1). [8] Mutation detection rates of *OPA1* in ADOA patients rangefrom 32% to 89%. [9-11] Aninsertion and deletion mutation in exon 28 (c.2826_2836delinsGGATGCTCCA) causing a frameshift has previously been shown to be common in Denmark due to a founder effect [12].

*OPA1* spans approximately 100 kb and is composed of 30 coding exons. Alternative splicing generates several isoforms. The main isoform is 960 amino acids long,encoded by 28 exons. The OPA1 protein is a ubiquitously expressed mitochondrial protein with similarity to dynamin-related GTPases.

Here we report the results of molecular genetic analyses of ADOApatients from Denmark. We identified two frequent mutations, besides the already known frequent founder mutation, and provide evidence for a founder effect in one and a probable mutational hotspot in the other. We report clinical data from families with the two new frequent mutations.

## Methods

### Patients

The cohort consisted of unrelated index patients from 93 families of Danish origin, clinically diagnosed with ADOA. In total 266 family members, 210 affected and 56 unaffected individuals were studied. Only cases from families with at least two affected members and a pattern compatible with autosomal dominant inheritance were included.

The diagnosis was based on routine clinical procedures, including refraction and determination of best-corrected visual acuity, color vision testing (various combinations of Farnsworth 100, Farnsworth-Roth 28, Americal Optical Hand-Hardy-Rittler, Farnsworth 15 Standard, Farnsworth 15 unsaturated, Lantony’sTritan album, and Nagel’s anomaloscope), direct ophthalmoscopy, slitlampbiomicroscopy of the anterior segment, vitreous, and posterior pole, fundus photography, Goldmann manual kinetic perimetry, and visual evoked potential recording. Clinical data from 19 families with one of two frequent mutations were retrospectively retrieved from a period of several decades.

The study followed the tenets of the Declaration of Helsinki and was approved by the local medical ethics committee (The Ethical Committee of Central Region (De VidenskabsetiskeKomiteer for Region Hovedstaden) H-B-2007-110). Patients and healthy relatives were informed of the nature of the study and written informed consent was obtained.

### Molecular genetic studies

Genomic DNA was extracted from leucocytes using Chemagic Magnetic Separation Module I (Chemagen, Baesweiler, Germany).

Analysis for c.2826_2836delinsGGATGCTCCA was performed by two separate PCR reactions using one common primer (OPA1R: TTG ATA GAC TAT AGG CAA GAA GAA) together with either a primer specific for the mutated allele (OPA1mutF: ACA GAG AAA GTG GAT GCT CC) or a primer specific for the wild type allele (OPA1wtF: CCA CAG AGA AAG TTA GAG AAA TTC). Fiftynanogram of genomic DNA was amplified in 30 μl volume using 10 pmol of each primer and AmpliTaq-Gold polymerase (Applied Biosystems, Foster City, CA, USA), with initial denaturation for 5 minutes at 95°C followed by 40 cycles of 95°C for 30 seconds, 56°C for 45 seconds and 72°C for 60 seconds, and a final extension at 72°C for 10 minutes. PCR products were analysed on agarose gel and ethidium bromide staining using standard methods.

DNA sequencing was performed of all 30 coding regions plus 20 bp of flanking sequences by PCR amplification using 50 ng of genomic DNA in a 50 μl volume using AmpliTaq Gold polymerase and 15 pmol each primer (see Additional file 1 for primer sequences). After initial denaturation at 95°C for 10 minutes, the samples were amplified for 35 cycles at 95°C for 30 seconds, 60°C for 30 seconds and 72°C for 30 seconds; final extension for 9 minutes at 72°C. Bidirectional sequencing was performed using BigDye v3.1 chemistry and ABI3130 (AppliedBiosystems). SeqScape software (version 2.5; Applied Biosystems) was used for analysis using reference sequence GenBank accession number NG_011605.1. GenBank transcript NM_015560 was used for numbering the variants with A in ATG as number 1.

Haplotype analysis was performed analysing 14 SNP’s surrounding the *OPA1* gene (position chr3:188,391,767–196,005,479, human genome version hg19/GRCh37) (Table 1). The analysis was performed by KBiosciences (Hoddesdon, Herts, United Kingdom). SNPs with minor allele frequency *>*25% in European descendents were selected for the study.

**Table 1:** Haplotype analysis

**A**											
Family DOA		139	141	142	143	144	145	146	147	148	140
Affected		1	7	3	2	6	6	11	2	3	3
Unaffected				1				2			
SNP	Position										
rs6796000	188,391,767	T	T	T	T	T	T	T	T	T	T
rs4677728	191,093,560	A	A	A	G	A	A	A	A	A	T
rs4453795	192,094,225	A	A	A	A	A	A	A	G	A	T
rs3905277	192,983,948	**A**	**A**	**A**	**A**	**A**	**A**	**A**	**A**	**A**	T
rs6788448	193,209,428	**C**	**C**	**C**	**C**	**C**	**C**	**C**	**C**	**C**	TC
rs11922359	193,290,262	**A**	**A**	**A**	**A**	**A**	**A**	**A**	**A**	**A**	A
rs9868128	193,299,458	**A**	**A**	**A**	**A**	**A**	**A**	**A**	**A**	**A**	TA
OPA1	193,310,933-193,415,599										
rs1007408	193,475,519	**C**	**C**	**C**	**C**	**C**	**C**	**C**	**C**	**C**	G
rs11915891	193,488,257	**C**	**C**	**C**	**C**	**C**	**C**	**C**	**C**	**C**	T
rs6770515	193,490,499	**A**	**A**	**A**	**A**	**A**	**A**	**A**	**A**	**A**	A
rs9854346	193,492,100	**A**	**A**	**A**	**A**	**A**	**A**	**A**	**A**	**A**	A
rs9866505	193,676,362	**C**	**C**	**C**	**C**	**C**	**C**	**C**	**C**	**C**	T
rs4677655	194,826,387	T	T	T	C	T	T	T	T	T	T
rs3772109	196,005,479	T	T	T	C	C	T	T	T	T	T
**B**											
Family DOA	Position	149	150	151	152	153	154	155	156	157	158
Affected		5	2	3	6	2	1	5	3	1	1
Unaffected			2		1	1		3	2		
SNP	Position										
rs6796000	188,391,767	T	T	T	T	T	T:T	T	T	T:T	T:T
rs4677728	191,093,560	A	A	A	A	A	G:A	G	G	A:A	G:A
rs4453795	192,094,225	G	GA	A	A	A	A:A	G	G	G:A	A:A
rs3905277	192,983,948	A	G	G	G	G	G:G	G	A	G:A	A:A
rs6788448	193,209,428	T	TC	TC	C	C	C:C	T	T	T:C	T:C
rs11922359	193,290,262	A	A	A	A	A	A:A	T	T	A:A	A:A
rs9868128	193,299,458	A	A	A	A	A	A:A	T	T	A:A	A:A
OPA1	193,310,933-193,415,599										
rs1007408	193,475,519	C	GC	GC	GC	G	G:C	GC	C	G:G	G:C
rs11915891	193,488,257	C	TC	T	T	TC	T:C	C	C	T:T	T:C
rs6770515	193,490,499	G	G	GC	G	GA	G:G	G	G	G:G	G:G
rs9854346	193,492,100	T	T	T	T	TA	T:T	T	T	T:T	T:T
rs9866505	193,676,362	C	TC	T	T	TC	T:C	C	C	T:T	T:C
rs4677655	194,826,387	TC	TC	TC	C	T	T:C	TC	T	T:T	T:C
rs3772109	196,005,479	T	TC	T	T	T	T:T	T	T	T:T	T:T

Position numbers according to hg19.

A) Analysis of 10 families with c.983A > G mutation in exon 9.

B) Analysis of 10 families with c.2708_2711delTTAG mutation in exon 27.

Investigation of c.1313A > C, c.1376 G > A and c.2496 G > C in a control group, was performed by searching the gene database generated by LuCamp (http://www.lucamp.org) by whole exome sequencing (average coverage of X 8) [13] of 1000 healthy Danish controls and 1000 Danish individuals with the combined phenotypes of type 2 diabetes, obesity and hypertension (Additional file 2).

## Results

Results from mutational analysis are shown in Table 2. The c.2826_2836delinsGGATGCTCCA mutation was found in 28 index patients. This mutation causes a frameshift and extension of the protein beyond the normal stopcodon (p. Arg943Aspfs*25). Sixty-five patients were left for further investigation. MLPA analysis showed deletions in 10 index patients, as previously reported. [14] In the remaining 55 index patients, DNA sequencing revealed a total of 24 sequence variations in 46 families including 15 novel mutations. The sequence variations were consisting of 16 nucleotide substitutions, six deletions, one insertion andtwo indels. On the protein level the sequence variations were predicted to cause three in-frame deletions, four nonsense mutations, fourmissense mutations, nine splice mutations and five frameshifts causing premature translation stop.

**Table 2:** Results of mutation analysis

**Mutation**	**Reference**	**Predicted change on protein level**	**Type**	**Exon/Intron**	**Domain**	**No of families (affected/unaffected)**
c.113_130del18	[15]	p. Arg38_Ser43del	Deletion (in-frame deletion)	Exon 2	Basic Domain	1 (2+/1-)
c.356_357delTT	Novel	p. Phe119X*	Deletion (nonsense)	Exon 3	Basic Domain	1 (2+/2-)
c.815 T > C	[16]	p. Leu272Pro	Substitution (missense)	Exon 8	GTPase Domain	1 (1+)
c.983A > G	[16]	p. Val291_Lys328del	Substitution (splice, deletion in-frame)	Exon 9	GTPase Domain	10 (43+/5-)
c.984 G > A	[11,16]	p. Lys328Lys	Substitution (splice)	Exon 9	GTPase Domain	1 (1+)
c.984 + 1 G > T	Novel	Unknown	Substitution (splice)	Intron 9	GTPase Domain	1 (1+)
c.1140 + 1 G > T	Novel	Unknown	Substitution (splice)	Intron 11	GTPase Domain	1 (1+)
c.1304_1305delGT	[15]	p. Cys435Tyrfs*9	Deletion (frameshift with premature stop)	Exon 13	GTPaseDomain	1 (4+)
c.1313A > C	Novel	p. Asp438Asp	Substitution (missense*)	Exon 14	GTPase Domain	1 (1+)
c.1376 G > A	Novel	p. Gly459Glu	Substitution (missense)	Exon 14	GTPase Domain	1 (1+/2-)
c.1544_1545delTA	Novel	p. Ile515Lysfs*4 (novel)	Deletion (frameshift with premature stop)	Exon 16	Dynamin Central Region	1 (1+/1-)
c.1665_1666insA	Novel	p. Met555Asnfs*7	Insertion (frameshift with premature stop)	Exon 17	Dynamin Central Region	1 (2+)
c.1687 C > T	Novel	p. Gln563X*	Substitution (nonsense)	Exon 17	Dynamin Central Region	1 (2+)
c.1983_1985delinsGG	Novel	p. Asp662Valfs*9	Deletion/insertion (frameshift with premature stop)	Exon 20	Dynamin Central Region	1 (1+)
c.2013 + 1 G > C	Novel	Unknown	Substitution (splice)	Intron 20	Dynamin Central Region	1 (2+)
c.2496 + 4_2496 + 5 delinsGTAAC	Novel	Unknown	Deletion/insertion (splice*)	Intron 24	Dynamin Central Region	1 (5+/1-)
c.2470 C > T	[17]	p. Arg824X*	Substitution (nonsense)	Exon 24	Dynamin Central Region	1 (1+)
c.2496 G > C	Novel	p. Leu832Phe/splice	Substitution (splice*)	Exon 24	Dynamin Central Region	1 (1+/3-)
c.2613 + 1 G > C	Novel	Unknown	Substitution (splice)	Intron 25		1 (2+/2-)
c.2614-9A > G	[18]	Unknown	Substitution (splice)	Intron 25		1 (7+)
c.2707 + 1 G > C	Novel	Unknown	Substitution (splice)	Intron26		4 (5+/1-)
c.2708_2711delTTAG	[2,9-11,15,16,19-21]	p. Val903Glyfs*3	Deletion (frameshift with premature stop*)	Exon 27		11 (29+/14-)
c.2713 C > T	[10]	p. Arg905X*	Substitution (nonsense)	Exon 27		1 (2+)
c.2728_2730 delGTT	Novel	p. Val910del	Deletion (in-frame deletion)	Exon 27		1 (8+/1-)
c.2826_2836delins GGATGCTCCA	[15,19]	p. Arg943Aspfs*25	Deletion/insertion (frameshift with extension)	Exon 28		28 (58+/18-)

*Based on silico analysis pathogenic. Chromatographs for novel mutations are shown in Additional file 4.

Fifteensequence variations were novel; of these, eleven introduced a premature stopcodon, caused deletion of an amino acid or were located in consensus splice sites. Threewere nucleotide substitutions, c.1313A > C, c.1376 G > A and c.2496 G > C. The c.1313A > C mutation is located as the first basepair in exon 14. *In silico* analysis using the splice site prediction of Berkeley Drosophila Genome Project (http://www.fruitfly.org/seq_tools/splice.html) predicts that c.1313A > C does not affect the splice site. Thus, this sequence variation probably causes a missense mutation p. Asp438Ala, predicted by SIFT (http://sift.jcvi.org/) to be damaging (SIFT score 0.01) and by Polyphen2 (http://genetics.bwh.harvard.edu/pph2/) to be probably damaging (Polyphen2 score 1.0). The c.1376 G > A variation is predicted to cause an aminoacid substitution, p. Gly459Glu, which is predicted to be pathogenic with SIFT score 0 and Polyphen2 score 1.0 and is probably a pathogenic missense mutation. Two unaffected family members did not have the variation.C.2496 G > C is the last basepair in exon 24 and is predicted by *in silico* analysis to destroy the donor splice site. Three unaffected family members did not have the variation. These three nucleotide substitutions no family members were available for segregation, however none were present in 1070 healthy Danish controls and 1000 Danish individuals with metabolic phenotypes (described under Materials and Methods section Molecular Genetic Studies). The indel c.2496 + 4_2496 + 5delinsGTAAC is located in intron 24. *In silico* analysis predicts that it destroys the splice site. The mutation segregated with the disease in this family (five affected and one unaffected).

Two sequence variations, c.983A > G and c.2708_2711delTTAG were present in 10 and 11 families, respectively. The c.983A > G mutation is reported to cause skipping of exon 9, resulting in an in-frame deletion of 38 amino acids. [16] The c.2708_2711delTTAG mutation is located as the first four nucleotides in exon 27. [2,9-11,15,16,19-21]*Insilico* analysis of the splice site predicts normal splicing; thus the sequence variation is predicted to cause a frameshift with incorporation of two amino-acids followed by a premature stop codon (p. Val903Glyfs*3). Haplotype analysis was performed using both affected and unaffected individuals from 10 families with c.983A > G and 10 families with c.2708_2711delTTAG (Table 1). Nine out of 10 families with c.983A > G shared the same haplotype of size 1,8 Mb while one family had a different haplotype distal to *OPA1*. At least two haplotypes were present for c.2708_2711delTTAG.

**Figure 1:**
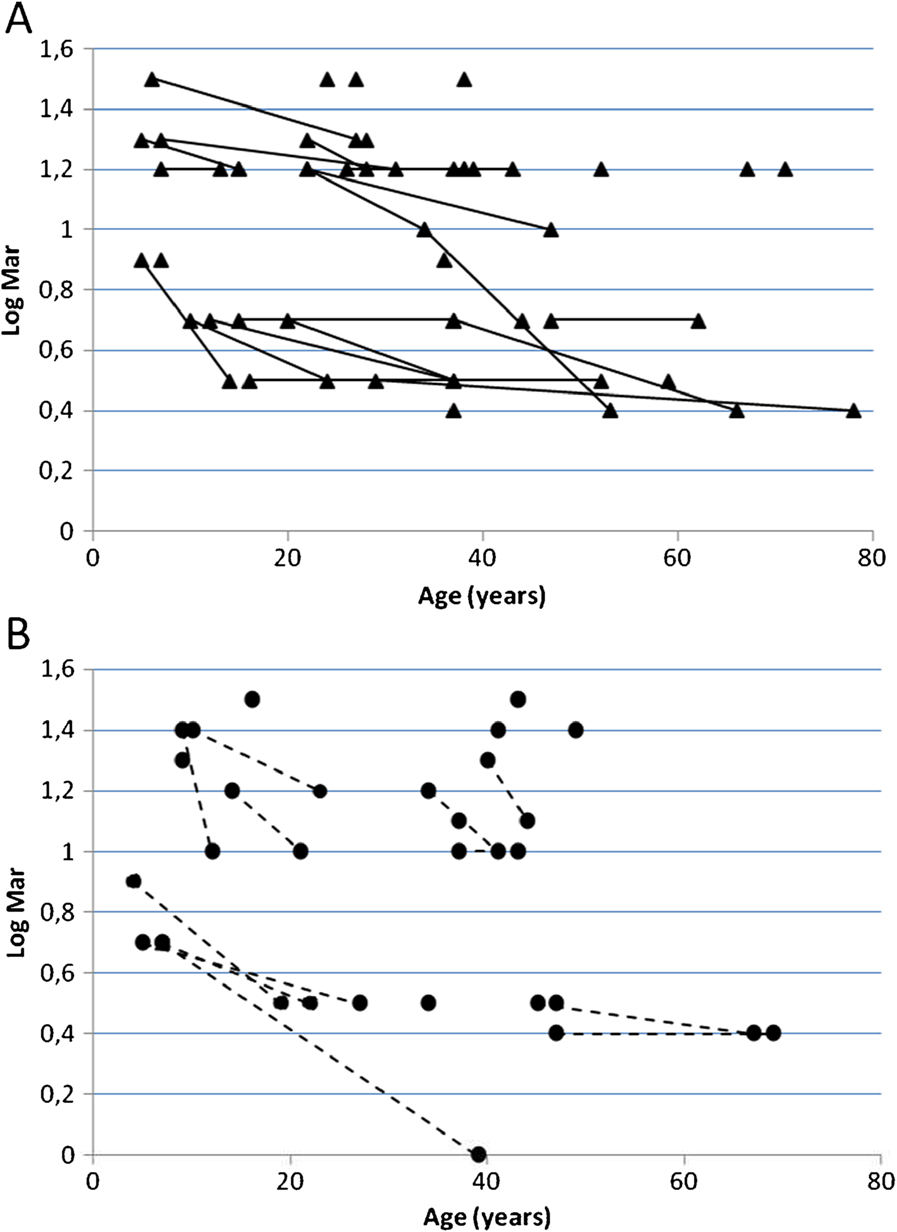
** Visual acuity converted from Snellen acuities into Log Mar.** Figure **A** includes 35 patients with c.983A > G mutation and figure **B** includes 21 patients with c.2708_2711delTTAG mutation.

Analysis of the clinical characteristicsof affected individuals with c.983A > G or c.2708_2711delTTAG revealed a broad spectrum of phenotype variation in both groups (Figure 1 and Additional file 3). For c.983A > G the age at clinical onset varied from 3 to 62 years of age (mean 13.8, median 7 years) while visual acuity ranged from 6/6 to 2/60. For c.2708_2711delTTAG the age at clinical onset varied from 3 to 48 years of age (mean 13.3, median 8 years) and visual acuity also ranged from 6/6 to 2/60. Figure 1 shows the visual acuities converted from Snellen acuities into Log Mar. A very large intrafamilial variation is evident and there is no obvious difference between the two groups of patients. The distribution seems to be dichotomous, one clusterhaving visual acuities 6/18 (Log Mar 0.5) or above, the other cluster with visual acuities 6/24 (Log Mar 0.6) or below. Among the two mutation groups 57% and 54% was in the best visual acuitycluster. There was no clear effect of age and in broad terms the full spectrum of variation in visual acuity was present throughout the age range that was surveyed. Patients for whom data from more than one examination was available (Figure 1) showed considerable variations in the rate of acuity loss. Colordiscrimination also exhibited large variations, tritan defects being common and with no correlation to visual acuity. Additionally, some patients had red-green defects of different magnitudes, whileothers had no detectable color vision defect. Full-field visual evoked potentials (ffVEP) were ofreduced amplitudes and somewhat prolongedlatencies with occasionalabnormal configurations, including “spike-trains”. Pattern VEP (pVEP) regularlypresented reduced or nearly undetectable amplitudes, but some patients had both normal ffVEP and pVEP even despite having moderately reducedvisual acuity. The most frequent clinical sign was temporal pallor of the optic discs.

## Discussion

In 84 of the 93 index patients (90%) we found sequence variations that were either obviously pathogenic or predicted to be pathogenic by *in silico* analysis. 53% were attributableto one of only threesequence variations (c.983A > G, c.2708_2711delTTAG, or c.2826_2836delinsGGATGCTCCA). This might explain the high prevalence 1:12,000 of Kjer-type optic atrophy in Denmark. The high mutation detection rate may reflect the clinical selection of the cohort from families with affected individuals in at least two generations and compatibility with autosomal dominant inheritance.

On the basis of the present study and the previously published MLPA study have lead us to perform mutational analysis in the most cost-effective manner: initially targeted analysis for c.2826_2836delinsGGATGCTCCA followed by MLPA analysis for deletions and duplications are performed. If these are negative, exon 9 and exon 27 are sequenced. In Danish patients this procedure can be expected to detect causative mutations in more than 60% of cases. Obviously, this strategy only applies to Danish ADOA patients, however, it must be emphasized that for other populations the fraction of deletions detectable by MLPA analysis and mutations detectable by DNA sequence analysis must be determined and probably both kinds of analyses must be performed to achieve a high detection rate.

In nine patients we did not detect pathogenic mutations. This might be due to mutations in regions not investigated (deep intronic mutations or mutations in regulatory regions) or mutations in other genes.

The two frequent mutations, c.983A > G and c.2708_2711delTTAG, have been reported previously in other ethnic groups. [2,9-11,15,16,19-21] Wefound evidence for a founder effect of c.983A > G while c.2708_2711delTTAG seems to be a mutational hotspot.

Clinical details of patients with c.983A > G mutation versus patients with c. 2708_2711delTTAG mutation revealed no obvious differences with respect to age at onset, visual acuity, refractive values, progression rate, color vision or VEP. In our study temporal pallor of the optical disc was the most constant clinical finding. This observation is in disagreement with the observations of Votruba et al. [22], who found a significant fraction of patients with a diffuse disc atrophy. The studydemonstrates no correlation between the included clinical parameters. The clinical data analysis is retrospective, however, and based on records collected over a long period where diagnostic practices may have varied. Nevertheless, thephenotype variation is conspicuous, even among patients with one and the same mutation. This allows for considerable optimism concerning the search for genes and environmental factors that modify the impact of *OPA1* mutations on the mitochondrial machinery and the associated phenotypes.

## Conclusions

Based on mutational analysis of Danish patients with ADOA we conclude that genetic testing for *OPA1*mutations assist in the diagnosis. Using a strategy based on MLPA analysis and DNA sequencing we have identified mutations in *OPA1* in 90% of families including 15 novel mutations. More than half of the affected families in Denmark are represented by three common mutations.

## Abbreviations

ADOA: Autosomal Dominant Optic Atrophy; B-Y: Blue- Yellow; Indel: insertion and deletion mutation; KB: Kilo base; L: Left; Mb: Mega bite; MLPA: Multiplex ligation-dependent amplification; ND: No Data; OMIM: Online Mendelian Inheritance in Man; SNP: Single nucleotide polymorphisms; Sph.eq: Spherical equivalent; TP: Temporal pallor of the optic disc; R: Right; R-G: Red-Green; VA: Visual acuity; pVA: Pattern visual acuity; ffVEP: Full-field visual evoked potentials; VEP: Visual evoked potentials.

## Competing interests

The authors declare that they have no competing interests.

## Authors’ contributions

All authors have read, edited and approved the final manuscript. GJA performed experiments, analyzed data and drafted the manuscript. KG helped designed the study, analyzed data and drafting the manuscript. JE and KG designed the study and experiments. KBN, ML, TR, HE contributed to writing of the paper. ML and TR helped with the ophthalmological clinical information.

## Pre-publication history

The pre-publication history for this paper can be accessed here:

http://www.biomedcentral.com/1471-2350/13/65/prepub

## Supplementary Material

Additional file 1 ***OPA1*primers used in mutation analysis.** Click here for file

Additional file 2 LuCamp partner list. Click here for file

Additional file 3 Clinical findings from families with c.983A > G and c.2708_2711delTTAG mutations. Click here for file

Additional file 4 Clinical findings from families with c.983A > G and c.2708_2711delTTAG mutations. Click here for file
