# Supplementary material for: Dominant optic atrophy in Denmark – report of 15 novel mutations in OPA1, using a strategy with a detection rate of 90%
Source: BMC Med Genet. 2012 Aug 2;13:65. doi: 10.1186/1471-2350-13-65 (PMC3507804; doi:10.1186/1471-2350-13-65)
Supplement: Additional file 1 — OPA1primers used in mutation analysis. [file 1471-2350-13-65-S1.pdf]

Exon/cdna segment	Forward primer (5'→3')	Inverse complement(5'→3')
1	acccactgcttactggcttatc<br>ctgctgagggccacttcct	gaggggcaaacaacagatggc<br>tcctgtcattctgggtcctc
2	acccactgcttactggcttatc<br>ttgctcttttaatgtcatttccttt	gaggggcaaacaacagatggc<br>catccaattgtattccactacacaa
3	acccactgcttactggcttatc<br>catgtttatttggcatgcag	gaggggcaaacaacagatggc<br>tttctctttcctcgagatgacc
4	acccactgcttactggcttatc<br>gggttgtcatgaggattaaacaa	gaggggcaaacaacagatggc<br>catgtatttttcctccatggttc
5	acccactgcttactggcttatc<br>aggctgttgacatcactgga	gaggggcaaacaacagatggc<br>tggatgtttttgtatttgcttctc
6	acccactgcttactggcttatc<br>aaaaatttaacttgctgtacattctg	gaggggcaaacaacagatggc<br>caccttccaaattttgctctg
7	acccactgcttactggcttatc<br>tcaagattttggaagattttaatttag	gaggggcaaacaacagatggc<br>cacacaacgttaagcggtaaaa
8	acccactgcttactggcttatc<br>caatgattatggaaaaacaatttga	gaggggcaaacaacagatggc<br>ttgcttaagacattacttggaaca
9	acccactgcttactggcttatc<br>aaaaactcagagcagcattacaaa	gaggggcaaacaacagatggc<br>tgaacaggtctcactgaagca
10 + 11	acccactgcttactggcttatc<br>gcaatgcagtagccctgtct	gaggggcaaacaacagatggc<br>gcagcaatctaaacatcaatacc
12 + 13	acccactgcttactggcttatc<br>gaatttttagaatacatttcaccaaaa	gaggggcaaacaacagatggc<br>tggattgctaaagaagaaaacat
14	acccactgcttactggcttatc<br>ttgctataatgtagacacaggggta	gaggggcaaacaacagatggc<br>tcacagctggagcttttaca
15 + 16	acccactgcttactggcttatc<br>ttttgctttctaaattgtatattacgc	gaggggcaaacaacagatggc<br>tgaaaacagttcaatttaagctactc
17	acccactgcttactggcttatc<br>tggtggtagtattgttgtccttttt	gaggggcaaacaacagatggc<br>atgccaaagattgccagcta
18	acccactgcttactggcttatc<br>aaccactacatctggaaagaagg	gaggggcaaacaacagatggc<br>agcttatcagatttttctctcaaca
19	acccactgcttactggcttatc<br>tctgaaaatcatgacagggtaaa	gaggggcaaacaacagatggc<br>caaggcaacaataaatcactgc
20	acccactgcttactggcttatc<br>tgatacttcagtcaagctgttttt	gaggggcaaacaacagatggc<br>cagctcctactcccttcaga
21	acccactgcttactggcttatc<br>tttttcatgttaaccattgaagtatg	gaggggcaaacaacagatggc<br>cctatgaaaaagtatcaatttgagaag
22	acccactgcttactggcttatc<br>ggaaggatatatttttatgctggttt	gaggggcaaacaacagatggc<br>ttggatgactccttcaccact
23	acccactgcttactggcttatc<br>cctttatttcaactgccttca	gaggggcaaacaacagatggc<br>tggtctagagccacaaaaagg
24	acccactgcttactggcttatc	gaggggcaaacaacagatggc

	caagcaccaaattatgaacca	tgcaagaaacaaatattaaggaagc
25	acccactgcttactggcttatc<br>tgtacaacttctcagtgtggttga	gaggggcaaacaacagatggc<br>gcatattttgacaactgttgctt
26	acccactgcttactggcttatc<br>cgatgatagttttcattttaactttgc	gaggggcaaacaacagatggc<br>tgggaagtattttggcatcc
27	acccactgcttactggcttatc<br>tctttattcatttataaaaacgatgc	gaggggcaaacaacagatggc<br>aaatgggaaaggtggaaagg
28	acccactgcttactggcttatc<br>cctcctgatttgtgatacctttg	gaggggcaaacaacagatggc<br>caagcaggatgtaaatgaagca
Alternativ 4b	acccactgcttactggcttatc<br>tgcccaattattgccctatc	gaggggcaaacaacagatggc<br>tgcaattttatttgcaactagtttatt
Alternativ 5b	acccactgcttactggcttatc<br>ctttgttgcacccttggttt	gaggggcaaacaacagatggc<br>cttggcagaaatggctgatt
