# Supplementary material for: Dominant optic atrophy in Denmark – report of 15 novel mutations in OPA1, using a strategy with a detection rate of 90%
Source: BMC Med Genet. 2012 Aug 2;13:65. doi: 10.1186/1471-2350-13-65 (PMC3507804; doi:10.1186/1471-2350-13-65)
Supplement: Additional file 2 — LuCamp partner list. [file 1471-2350-13-65-S2.pdf]

LuCamp partner list

Professor, LuCamp Centre Director, Oluf B. Pedersen
1) Hagedorn Research Institute, Gentofte, Denmark
2) Marie Krogh Center for Metabolic Research, Section of Metabolic Genetics,
Faculty of Health Sciences, University of Copenhagen, Denmark

MD, PhD, Research Manager, Daniel R. Witte
Steno Diabetes Center, Gentofte, Denmark.

Professor Torben Hansen
1) Marie Krogh Center for Metabolic Research, Section of Metabolic Genetics,
Faculty of Health Sciences, University of Copenhagen, Denmark
2) Faculty of Health Sciences, University of Southern Denmark, Odense, Denmark.

Ph.d. Gitte Andersen
Hagedorn Research Institute, Copenhagen, Denmark.

Professor Jun Wang
BGI-Shenzhen, Shenzhen, China

Professor Lars Bolund
Department of Human Genetics, University of Aarhus, Denmark

Professor Torsten Lauritzen
Faculty of Health Sciences, University of Aarhus, Denmark

Professor, Head of Department, Karsten Kristiansen
Department of Biology, University of Copenhagen, Denmark

Professor Torben Jørgensen
Research Centre for Prevention and Health, Glostrup University Hospital, Glostrup, Denmark.

Professor Arne Astrup
Department of Human Nutrition, Faculty of Life Sciences, University of Copenhagen,
Copenhagen, Denmark.

Professor Thue W. Schwartz
1) Marie Krogh Center for Metabolic Research, Metabolic Receptology and
Enteroendocrinology
Faculty of Health Sciences, University of Copenhagen, Denmark
2) Laboratory for Molecular Pharmacology, University of Copenhagen, Copenhagen,
Denmark

Professor Rasmus Nielsen
1) Department of Biology, University of Copenhagen, Copenhagen, Denmark
2) Department of Statistics, University of California Berkeley, Berkeley, California, USA.

Ph.d. Anders Albrechtsen
1) Department of Biology, University of Copenhagen, Copenhagen, Denmark
2) Department of Statistics, University of California Berkeley, Berkeley, California, USA.
