# Supplementary material for: Dominant optic atrophy in Denmark – report of 15 novel mutations in OPA1, using a strategy with a detection rate of 90%
Source: BMC Med Genet. 2012 Aug 2;13:65. doi: 10.1186/1471-2350-13-65 (PMC3507804; doi:10.1186/1471-2350-13-65)
Supplement: Additional file 3 — Clinical findings from families with c.983A > G and c.2708_2711delTTAG mutations. [file 1471-2350-13-65-S3.pdf]

Family and Patient ID	Age at Onset	VA<br>R<br>L (Age)	VA<br>R<br>L (Age)	Refraction R/L sph.eq.	Fundus	Color vision	VEP flash	VEP checkerboard
**Exon 9; c.938A>G**								
139a	7	6/36<br>6/24 (12)	6/60<br>6/60 (37)	-3.5/-4.5	TP	Mild R-G defect	ND	ND
140a	4	6/24<br>6/24 (5)	6/60<br>6/36 (14)	Emmetropia	TP	Mild R-G and<br>B-Y defect	Spike trains	Normal
140c	14	6/36<br>3/36 (37)	3/36<br>3/60 (66)	+1.5/+5.5	TP	Moderate defect without specified axis	Reduced amplitude, prolonged latency	Severely reduced
140d	ND	6/60<br>6/60 (29)	3/60<br>3/60 (78)	Emmetropia	TP	ND	Reduced amplitude, severely reduced	ND
140e	7	3/60<br>3/60 (53)		-6.25/-4.75	TP	ND	Reduced amplitudes, normal latency	Reduced amplitude, normal latency
140f	7	6/9<br>6/6 (7)	6/12<br>6/12 (31)	-0.5/-0.25	TP	Moderate defect without specified axis	ND	Normal
140g	7	6/36<br>6/18 (16)	6/60<br>6/36 (52)	Emmetropia	TP	ND	Spike trains	Spikes and prolonged latency
141b	7	6/24<br>6/36 (36)		-2.5/-2.5	TP	Mild R-G and<br>B-Y defect	Normal	Severely reduced amplitudes
141d	29	6/9<br>6/9 (22)	6/12<br>6/36 (28)	Emmetropia/<br>-0.75	Normal	Normal	Normal	Normal
141e	ND	6/36<br>6/60 (44)		+0.5/+11.25	TP	Moderate R-G and<br>B-Y defect	ND	ND
142a	22	6/12<br>6/12 (22)	6/18<br>6/12 (47)	+1.0/Emmetropia	TP	Mild R-G and<br>strong B-Y defect	Spikes, prolonged latency	Reduced amplitudes, normal latency
142b	ND	6/12<br>6/12 (71)		+2.0/+1.75		Moderat R-G defect		ND
143a	62	6/12<br>6/18 (67)		+1.25/+0.75	TP	Mild R-G and<br>B-Y defect	Spikes, prolonged latency	Reduced amplitudes, normal latency
143b	ND	6/6<br>6/6 (38)		ND	Normal	ND	ND	ND
143c	ND	6/12<br>6/9 (38)		Emmetropia	Normal	ND	ND	ND
143d	3	6/24<br>6/18 (7)		+2.5/+2.5	Normal	Normal	ND	ND
143e	10	6/12<br>6/12 (15)		-1.25/-0.75	TP	Mild B-Y defect	Normal	Reduced amplitudes, normal latency
144a	18	6/12<br>6/18 (22)	6/18<br>6/24 (34)	-1.25/-1.25	TP	Mild B-Y defect	Normal	Reduced amplitudes
144b	7	6/12<br>6/12 (26)	6/12<br>6/12 (38)	Emmetropia	TP	Normal	Normal	Severely reduced amplitudes
144c	5	6/36<br>6/60 (15)	6/36<br>3/60 (37)	-0.5/-0.5	TP	Moderat B-Y defect	Normal	Normal
144d	7	3/60<br>3/60 (37)		-0.75/-1.25	TP	Normal R-G and<br>strong B-Y defect	Normal	ND

144f	ND	6/6<br>6/6 (6)	6/9<br>6/9 (27)	+2.5/+2.0	Normal	ND	ND	ND
145a	52	6/12<br>6/12 (52)		-1.5/-1.25	TP	Moderate R-G and strong B-Y defect	Normal	Normal
145b	13	6/60<br>6/60 (59)		-1.5/-0.5	TP	Strong R-G and B-Y defect	Normal	Normal
145c	6	6/36<br>6/36 (20)	6/60<br>3/60 (37)	Emmetropia	TP	Moderate R-G and B-Y defect	Prolonged latency	Severely reduced amplitudes
145d	ND	6/6<br>6/6 (27)		Emmetropia	Normal	ND	ND	ND
145e	4	6/36<br>6/36 (10)	6/60<br>6/36 (24)	-2.0/-2.25	TP	Moderate R-G defect	Normal	Reduced amplitudes
145g	ND	6/12<br>6/36 (39)	6/12<br>6/36(43)	-3.25/-2.75	TP	Mild R-G and strong B-Y defect	Normal	Reduced amplitudes
145h	7	6/36<br>6/36(47)	6/36<br>6/36 (62)	-1.25/-1.0	TP	ND	Reduced amplitudes, prolonged latency	Reduced amplitudes
145j	5	6/9<br>6/9(5)	6/12<br>6/12 (15)	Emmetropia	Normal	Normal	Reduced amplitudes	Reduced amplitudes
145k	7	6/12<br>6/18 (7)	6/12<br>6/18 (13)	+1.0/+1.5	TP	Mild R-G defect	Normal	Normal
145l	ND	6/6<br>6/6 (24)		Emmetropia	normal	Mild R-G and B-Y defect	Reduced amplitudes	Reduced amplitudes
145m	14	6/9<br>6/9 (28)		-4.5/-4.5	TP	Mild R-G and B-Y defect	Normal	Severely reduced amplitudes
146a	28	6/24<br>6/18 (34)	3/60<br>2/60 (53)	-4.25/-4.0	TP	Mild R-G and strong B-Y defect	ND	ND
147b	7	6/12<br>6/12 (37)		-3.0/-3.5	TP	Moderat B-Y defect	Reduced amplitudes	Reduced amplitudes
**Exon 27; c.2708_2711delTTAG**								
149b	5	6/36<br>6/36 (5)		Emmetropia	TP	Moderate R-G and strong B-Y defect	Prolonged latency, Spike trains	Severely reduced amplitudes
149c	3	6/36<br>6/36 (7)	2/60<br>2/60 (39)	Emmetropia	TP	Strong R-G and B-Y defect	Reduced amplitudes, Spike trains	ND
149d	No symptoms	6/7.5<br>6/7.5 (41)		-5.5/-7.0	Myopic TP	Moderate defect without preferential axis	Normal	ND
150a	4	6/36<br>6/36 (7)	6/60<br>6/60 (22)	Emmetropia	TP	Moderate R-G and B-Y defect	ND	ND
150c	8	6/18<br>6/24 (37)	6/24<br>6/24 (43)	-4.5/-5.25	TP	Mild R-G and B-Y defect	ND	ND
151a	5	6/36<br>6/36 (7)		+1.5/+1.5	TP	Normal	Normal	Severely reduced amplitudes
151c	7	6/60<br>6/60 (34)		Emmetropia	TP	ND	ND	ND
152a	7	3/36<br>2/36 (47)	3/60<br>1/36 (69)	-1/Emmetropia	TP	ND	Prolonged implicit times, Spike trains	Normal
152b	4	6/7,5<br>6/7,5 (9)	6/18<br>6/18 (12)	-1/-0.75	TP	Normal R-G and mild B-Y defect	Normal	Severely reduced amplitudes
152c	8	6/6<br>6/7.5 (16)		+0.5/+0.5	TP	Normal	Spike trains	Moderately reduced amplitudes

152d	18	6/60<br>2/60 (45)	3/60<br>2/60 (67)	-5.75/-5.75	TP	Moderate	ND	ND
152e	43	6/6<br>6/6 (43)		-4/-4.25	Myopic	Normal	Slightly prolonged latency	Severely reduced amplitudes
152f	33	6/12<br>6/60 (34)	6/18<br>6/60 (41)	+2.25/+5.25	TP	Mild R-G and B-Y defect	Normal	Reduced amplitudes
153a	6	6/60<br>6/60 (47)		-0.75/-0.75	TP	Anamnestically reduced	ND	ND
154a	4	6/24<br>6/24 (4)	6/60<br>6/60 (19)	-1/-1	TP	Strong R-G and B-Y defect	Normal	Severely reduced
155c	48	6/7.5<br>6/9 (49)		-2.5/-0..5	TP	Moderate R-G defect	Normal	Severely reduced
156a	10	6/7.5<br>6/7.5 (10)	6/12<br>6/12 (23)	-7/-6.5	TP	Normal	Normal	Normal
156d	9	6/9<br>6/6 (9)	6/9.5<br>6/9.5 (8)	Emmetropia	Normal optic discs	Normal	Normal	Reduced amplitudes
156e	11	6/9<br>6/9 (40)	6/15<br>6/15 (44)	Emmetropia	TP	Strong B-Y defect	ND	ND
157a	18	6/15<br>6/30 (37)		Emmetropia	TP	Normal	Normal	Slightly reduced amplitudes, slightly prolonged latency
158a	14	6/12<br>6/12 (14)	6/18<br>6/24 (21)	-1/-1	TP	Strong R-G and B-Y defect	Reduced amplitudes, Spike trains	Normal

TP: Temporal pallor of the optic disc; R: Right; L: Left; VA: Visual Acuity; Sph.eq.: Spherical equivalent; VEP: Visual evoked Potentials; R-G: Red-Green; B-Y: Blue-Yellow; ND: No data availably
