# Supplementary material for: Dominant optic atrophy in Denmark – report of 15 novel mutations in OPA1, using a strategy with a detection rate of 90%
Source: BMC Med Genet. 2012 Aug 2;13:65. doi: 10.1186/1471-2350-13-65 (PMC3507804; doi:10.1186/1471-2350-13-65)

Additional file 4: Chromatographs of the 15 novel mutations

Exon 3: c.356_357delTT

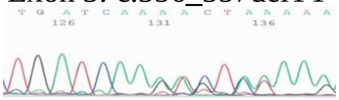

Intron 9: c.984+1G>T

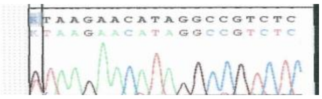

Intron 11: c.1140+1G>T

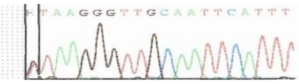

Exon 14: c.1313A>C

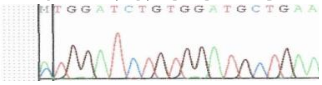

Exon 14: c.1376G>A

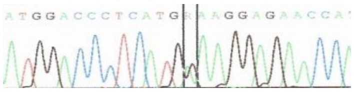

Exon 16: c.1544_1545delTA

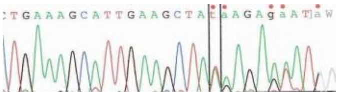

Exon 17: c.1665_1666insA

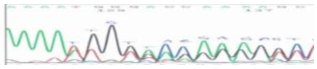

Exon 17: c.1687C>T

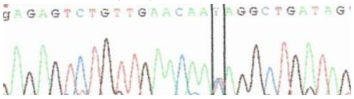

Exon 20: c.1983_1985delinsGG

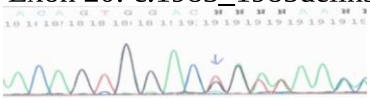

Intron 20: c.2013+1G>C

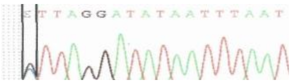

Intron 24: c.2496+4_2496+5delinsGTAAC

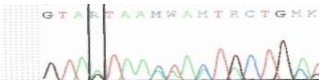

Exon 24: c.2496G>C

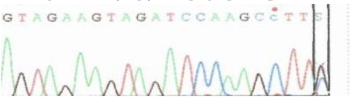

Intron 25: c.2613+1G>C

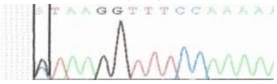

Intron 26: c.2707+1G>C

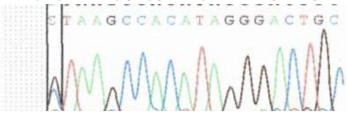

Exon 27: c.2713C>T

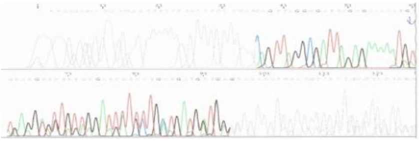

Supplement: Additional file 4 — Clinical findings from families with c.983A > G and c.2708_2711delTTAG mutations. [file 1471-2350-13-65-S4.pdf]
